# Generalized anxiety disorder among rural primary and middle school students during the outbreak of COVID-19: a multicenter study in three southern Chinese cities

**DOI:** 10.1186/s12889-023-15215-8

**Published:** 2023-02-14

**Authors:** Zidan Yang, Yongxin Zhang, Haijie Xu, Menglai Gan, Jianrui Ma, Jiarong Liu, Xiner Tan, Wenjing Hou, Wanbao Ye, Liping Li

**Affiliations:** 1 School of Public Health, Shantou University, Shantou, Guangdong Province China; 2 Injury Prevention Research Center, Shantou University Medical College, Shantou, Guangdong Province China; 3 Cancer Hospital of Shantou University Medical College, Shantou, Guangdong Province China; 4 Shantou University Medical College, Shantou, Guangdong China

**Keywords:** Anxiety, COVID-19, Rural areas, Psychological impact, Coronaphobia

## Abstract

**Background:**

The major public health crisis caused by the rapid spread of the coronavirus disease 2019 (COVID-19) and the large-scale public health measures such as social isolation and school closures enforced by some countries have severely affected on the physical and mental wellbeing of children and adolescents globally. This study aimed to estimate the prevalence of the psychological impact and investigate the similarities and differences in the influential factors for generalized anxiety disorder among rural adolescents as a relatively lesser noticed population the outbreak of COVID-19.

**Methods:**

From May 11 to 22, 2020, a total of 1,179 adolescents, including Grade 5–6 in primary school and Grade 7–8 in middle school, were selected by multistage sampling in three Southern Chinese cities (Shantou, Guangdong Province; Hezhou, Guangxi Province; Nanchong, Sichuan Province), and completed the questionnaires including sociodemographic, generalized anxiety disorder, academic stress, coronaphobia, knowledge of COVID-19, and precautionary measures. ANOVA, Chi-square test, Kruskalwallis H test and multivariate linear regression were performed in the statistical analysis.

**Results:**

The average scores of generalized anxiety disorder during the past two weeks were 3.43 (SD 4.46), 4.47 (SD 5.15), and 4.10 (SD 4.94) in Shantou, Hezhou and Nanchong, respectively. For the pooled data, academic stress (*P* < 0.001), coronaphobia (*P* < 0.001), and precautionary measures (*P* = 0.002) contributed to the prediction of anxiety scores. Academic stress was significantly associated to anxiety symptoms in all cities (*P* all < 0.001). Coronaphobia was also significantly associated to anxiety symptoms in all cities (*P* all < 0.001).

**Conclusion:**

This study highlights the urgent need for researchers and policymakers to focus on the mental health of rural children and adolescents during the COVID-19 epidemic. The adolescents with academic stress and coronaphobia, the greater the risk that adolescents will suffer from anxiety, suggesting mental health counseling and professional family support are needed.

## Background

Since December 2019, the coronavirus disease 2019 (COVID-19) has been spreading at an alarming rate in several countries around the world, including China, posing a major threat to public health worldwide and becoming one of the major public health problems [1, 2]. Less than five months after its emergence, millions of people worldwide have been infected asymptomatically or symptomatically, and the number of infections continues to grow [3]. To curtail the further spread of COVID-19 and prevent potential large-scale outbreaks, many regions in China initiated Level 1 responses and have adopted unprecedented social distancing and quarantine measures [2, 4]. While quarantine or lockdown situations were effective [5], unlike normal routine situations, the measures would expose people to more widespread and complex stressors [6]. Until early May of the same year, the public health emergency response was lowered to less than Level 2 throughout China [7].

In China, mental health problems have been more common in rural areas than in urban areas [8, 9]. Under the COVID-19 pandemic, the scale, rapid development, and impact of large-scale regional containment were historically unprecedented, as were the changes in the physical, psychological, and social conditions of the rural population in this status. In other words, the pandemic has significant mental health and community implications in rural social relations [6]. Therefore, dealing with mental health problems of people living in rural areas has become an issue that cannot be ignored.

Adolescence is a vulnerable stage during which adolescents go through a difficult transition, making them particularly vulnerable to the adverse effects of COVID-19 [10, 11]. In particular, primary and middle school students, being a relatively young group of adolescents, are more susceptible than the general population to psychiatric disorders, such as anxiety, depression, substance use disorders, eating disorders, and psychosis [12]. Following the COVID-19 outbreak, schools across the country were asked by the Ministry of Education to temporarily close and students were requested to stay at home. Decreased social interaction, difficulties in schoolwork, restrictions on staying at home, fear of sickness, substantial changes in daily routine and boredom might create tremendous psychological impacts on adolescents. They were more susceptible to the psychological effects of the outbreak and were weak in dealing with their psychological distress [13]. Failure to address psychological issues promptly might lead to poor health, education and economic situations at a later date [14].

Although COVID-19 spreads more rapidly in big cities, the mental health effect of COVID-19 on rural adolescents cannot be disregarded, as they deserve the support of intervention and prevention strategies. Thus, this multicenter study focused on a seldom researched sample of rural primary and middle school students who were harder to reach but might be affected by the pandemic as much or more. We hypothesized that factors associated with the COVID-19 outbreak have an influential role in rural students’ anxiety. We investigated their anxiety status after school reopening (mid-May 2020) in Southern China and further examined the relationship between anxiety status and possible factors such as demographic, academic stress, coronaphobia, knowledge, and precautionary measures during the sensitive period.

## Method

### Participants

**Fig. 1:**
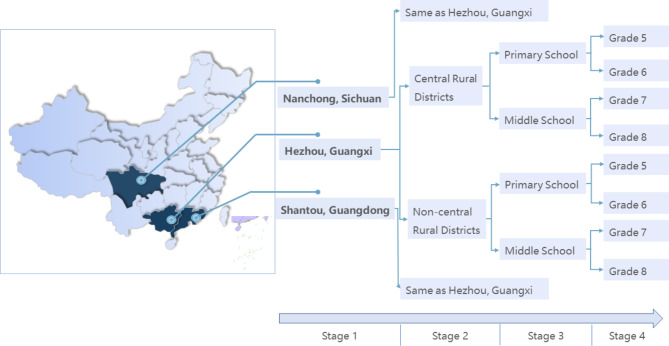
Three undeveloped cities in southern China selected by multiple-stage cluster sampling

A multicenter cross-sectional study was conducted to examine the psychological status of rural students utilizing a field questionnaire from May 11 to 22, 2020. Participants in southern China were selected through stratified cluster sampling. First, we selected three undeveloped cities in southern China by multiple-stage cluster sampling (Shantou, Guangdong; Hezhou, Guangxi; Nanchong, Sichuan). Second, in the central and non-central regions of the above three rural areas, one primary school and one secondary school were selected respectively, for a total of 12 schools. Third, students from two classes of each grade, including Grade 5–6 in primary school and Grade 7–8 in middle school (Fig. 1), were invited to complete a uniform questionnaire. The following inclusion criteria were used: (1) studied and lived in the investigated districts during the outbreak; (2) able to read and understand the questionnaire; (3) had not been diagnosed with a mental illness other than mild, moderate and severe anxiety, or taking medications for mental illness; (4) written informed consent. They could be included only if all conditions were met. A sample size was calculated considering a 20% expected prevalence, a margin of error of 5%, a confidence level of 95%, and a rejection rate of 20%. Therefore, at least 270 participants should be surveyed.

### Data collection

The investigators were trained prior to the survey. We obtained consent from the relevant heads of schools for on-site support and organizational coordination. In schools, the teachers of the study site classes have been informed in advance of the purpose and requirements of our questionnaire and utilized the class meetings to assist the investigators in asking students to complete the questionnaire independently. And the investigators took it back after on-site verification. We sent out the questionnaires to 1204 students. After excluding 25 invalid questionnaires which were unfinished or finished but with too much missing data (over 20% of the total items), a total of 1179 participants were finally eligible to enroll in the study. The response rate of this study was 97.92%. Before filling out the questionnaire, participants voluntarily signed an informed consent form. This study was approved by the ethics committee of Shantou University Medical College.

Double entry verification was used to maintain the correctness and accuracy of the data. The median or mean was used as a supplement if there were missing data entries.

### Measurements

#### Socio-demographic characteristics

Basic information obtained from the respondents included age, gender, grade, parental educational level, parental occupations, household economic status, and whether lives with parents.

#### Anxious symptoms

The Generalized Anxiety Disorder 7-Item (GAD-7) Scale in Chinese is currently one of the most widely used tools for detecting and assessing anxiety disorders in China due to its satisfactory reliability and efficiency [15, 16]. The individuals rated how often they experienced symptoms of generalized anxiety disorder (e.g., “Becoming easily annoyed or irritable”) on a scale of 0 (never) to 3 (almost daily for the past two weeks). The scores of GAD-7 (ranging from 0 to 21) showed good psychometric properties in adolescent samples [17, 18], with higher scores indicating more severe anxiety symptoms. A cutoff value of GAD-7 ≥ 4 was used to screen for anxiety symptoms [19].

#### Academic stress

The modified Academic Stress Scales [20] was used to assess academic perceived stress. The participants were asked how frequently they had certain thoughts and feelings in learning (e.g., " I worry about what others think of my academic performance”) in the last two weeks. Participants were made on a three-point scale: “1 = never,” “2 = occasionally,” “3 = often”. We calculated scores for the items, with higher scores indicating higher levels of academic stress.

#### Fear of contracting COVID-19 (Coronaphobia)

Coronaphobia, a new emerging phobia specific to COVID-19 [21], was measured using Lee’s 5-item scale(2020) [22, 23]. The respondents were requested to rate the frequency of coronavirus anxiety disorder symptoms (e.g., “I felt torpid or stiff when thinking about or exposed to information of coronavirus”) in the last two weeks on a 5-point scale ranging from 0 (not at all) to 4 (almost every day).

#### Knowledge of COVID-19

The knowledge questionnaire was based on the prevention and control guidelines regarding COVID-19 (5th Edition) being propagated by the Chinese Center for Disease Control and Prevention [24]. Overall, four questions were on disease manifestation and severity (K1-K4), five were on disease transmission (K5-K9), and five were on disease prevention and control (K10-K14). A correct answer was scored 1, and an incorrect/unknown answer was 0. All 14 answers were summarized to generate a total COVID-19 knowledge score from 0 to 14 for each participant.

#### Precautionary measures

We selected several types of preventing behaviors from the COVID-19 prevention guidelines from WHO [25] and understood the situation of students taking the initiative to obtain COVID-19 information. Such behaviors included (1) washing hands correctly, (2) wearing a mask when going out, (3) staying home as much as possible, (4) exercising regularly, (5) trying to eat well-balanced and healthy meals, (6) coughing into a bent elbow or tissue, and (7) obtaining epidemic information proactively. We calculated average scores for these seven items, in which higher scores represented higher participation in preventive behaviors.

### Data analysis

Descriptive statistics were used to describe the respondents’ sociodemographic characteristics. Qualitative variables were presented as n (%), and quantitative data as mean ± standard deviation. ANOVA, Chi-square test as well as Kruskal-Wallis H test were used to assess the significance of the above factors among three cities. Anxiety score was used as dependent variables and sociodemographic characteristics, academic stress, coronaphobia, knowledge of COVID-19, and precautionary measures were used as independent variables. A multivariate linear regression was performed after an assessment for collinearity, in order to assess the significance between anxiety score and the independent variables. Standard multivariate linear regressions (adjusted models), controlling for demographic (i.e., gender and grade) by including fixed effects, were further used to analyze the significant factors in the above regression. In all analyses, a two-sided *P* value of < 0.05 was considered statistically significant. IBM SPSS version 26.0 was used for summary statistics and tests whereas R 4.0.2 was used to generate the graph presented. Moreover, PowerPoint 2019 was used to create geographic map.

## Results

### Sample and sociodemographic statistics

Table 1 showed the main sociodemographic characteristics of the respondents aged 10–16 years by city (47.41% male, mean age 12.83 (SD 1.27)). Among them, male accounted for 45.14% in Shantou, 43.72% in Hezhou and 53.04% in Nanchong. The mean age of the participants in Shantou, Hezhou and Nanchong were 13.46 ± 1.19 years, 12.76 ± 1.17 years and 12.33 ± 1.21 years, respectively. More than half of the students in Shantou and Nanchong had a primary school education. The education levels of the participants in Hezhou were Primary school (43.72%).

**Table 1:** Demographics of Respondents of Rural Primary and Middle School Students in Southern China

Variables, *n (%)*	Overall (*n* = 1179)	Shantou, Guangdong 370 (31.38)	Hezhou, Guangxi 398 (33.76)	Nanchong, Sichuan 411 (34.86)	*χ*^*2*^ / *F*	*p*
**Age (Years)**	12.83 ± 1.27	13.46 ± 1.19	12.76 ± 1.17	12.33 ± 1.21	88.77^a^	< 0.001
**Gender**					8.17^b^	0.017
Male	559(47.41)	167(29.87)	174(31.13)	218(39.00)		
Female	620(52.59)	203(32.74)	224(36.13)	193(31.13)		
**Study stage**					9.19^b^	0.010
Primary school	641(54.37)	201(31.36)	195(30.42)	245(38.22)		
Secondary school	538(45.63)	169(31.41)	203(37.73)	166(30.86)		
**Father’s education level**					7.20^c^	0.027
Under junior high school	674 (57.17)	186(27.6)	237(35.16)	251(37.24)		
Senior school	172 (14.59)	71(41.28)	41(23.84)	60(34.88)		
Undergraduate	30 (2.54)	16(53.34)	7(23.33)	7(23.33)		
Master degree or above	303 (25.70)	97(32.01)	113(37.30)	93(30.69)		
**Mother’s education level**					1.57^c^	0.456
Under junior high school	691 (58.61)	205(29.67)	235(34.01)	251(36.32)		
Senior school	132 (11.20)	52(39.40)	36(27.27)	44(33.33)		
Undergraduate	24 (2.03)	14(58.33)	3(12.50)	7(29.17)		
Master degree or above	332 (28.16)	99(29.82)	124(37.35)	109(32.83)		
**Father’s occupation**					152.02^b^	< 0.001
Health worker	46 (3.90)	25(54.35)	10(21.74)	11(23.91)		
Non-medical institute staff	185 (15.69)	88(47.57)	43(23.24)	54(29.19)		
Business and service practitioner	408 (34.61)	51(12.50)	209(51.23)	148(36.27)		
Migrant worker and farmers	196 (16.62)	61(31.12)	44(22.45)	91(46.43)		
Other	344 (29.18)	145(42.15)	92(26.74)	107(31.1)		
**Mother’s occupation**					128.44^b^	< 0.001
Health worker	46 (3.90)	20(43.48)	14(30.43)	12(26.09)		
Non-medical institute staff	181 (15.35)	82(45.30)	48(26.52)	51(28.18)		
Business and service practitioner	380 (32.23)	44(11.58)	195(51.32)	141(37.10)		
Migrant worker and farmers	262 (22.22)	99(37.78)	69*26.34)	94(35.88)		
Other	310 (26.30)	125(40.32)	72(23.23)	113(36.45)		
**Whether lives with parents**					265.44^b^	< 0.001
Father only	86 (7.30)	8(9.30)	46(53.49)	32(37.21)		
Mother only	127 (10.77)	21(16.53)	32(25.20)	74(58.27)		
Both parents	725 (61.49)	316(43.59)	269(37.10)	140(19.31)		
Neither	241 (20.44)	25(10.37)	51(21.16)	165(68.47)		
**Low-income family**					41.51^b^	< 0.001
Yes	146 (12.38)	20(13.70)	75(51.37)	51(34.93)		
No	551 (46.74)	165(29.95)	176(31.94)	210(38.11)		
Unknown	482 (40.88)	185(38.38)	147(30.50)	150(31.12)		

**Note.**^a^ ANOVA for differences by city

^b^ Chi-square test for differences by city

^c^ Kruskal-Wallis H test for differences by city

**Fig. 2:**
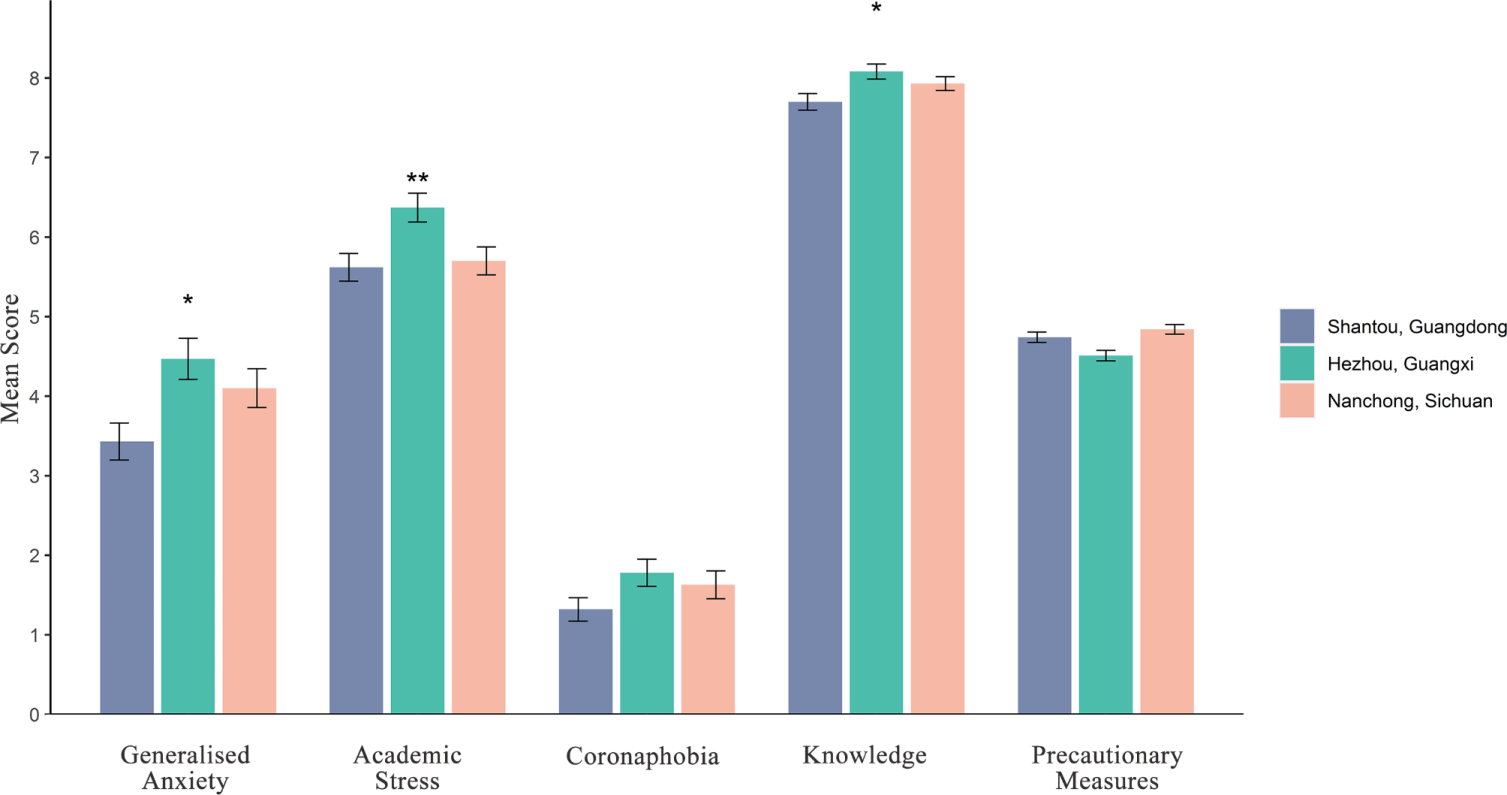
The Average Scores of Generalized Anxiety, Academic Stress, Coronaphobia, Knowledge and Precautionary Measures of Rural Students in Southern China Note. Statistical significances by city were represented in figure by asterisks as follows: * < 0.05; ** < 0.01

The average scores of generalized anxiety, academic stress, coronaphobia, knowledge and precautionary measures of COVID-19 in different rural areas were shown in Fig. 2. Significant differences between rural areas were found in generalized anxiety, academic stress and knowledge. The average generalized anxiety score in the past two weeks varied from a low of 3.43 (SD 4.46) in Shantou to a high of 4.47 (SD 5.15) in Hezhou. The average scores of academic stress and knowledge were highest in Hezhou, followed by Nanchong, and lowest in Shantou.

### Linear regression analysis

Table 2 showed the linear regression results of the total generalized anxiety scores of the three rural areas in the study. Demographics of participants were not statistically associated with generalized anxiety scores, with the exception of grade and region. Academic stress was associated with higher generalized anxiety scores (*P* < 0.001). The coronaphobia scores varied positively and significantly (*P* < 0.001), and the precautionary measures scores varied inversely (*P* = 0.002) with generalized anxiety scores.

**Table 2:** Analysis of the Influencing Factors of Generalized Anxiety

	Generalized anxiety		
Variates	Coefficients (95.0% CI)	*t*	*P*
**Gender**	0.34(-0.20 to 0.88)	1.246	0.213
**Grade**	-0.26(-0.52 to 0.00)	-1.977	0.048
**Shantou**	-0.78(-1.51 to -0.04)	-2.083	0.038
**Hezhou**	-0.45(-1.12 to 0.21)	-1.332	0.183
**Father’s education level**	-0.06(-0.40 to 0.27)	-0.372	0.710
**Mother’s education level**	0.15(-0.18 to 0.47)	0.889	0.374
**Father’s occupation**	-0.20(-1.51 to 1.10)	-0.306	0.759
**Mother’s occupation**	-0.20(-1.60 to 1.19)	-0.288	0.773
**Whether lives with parents**	0.15(-0.21 to 0.51)	0.810	0.418
**Low-income family** ^**#**^	-0.09(-0.76 to 0.58)	-0.269	0.788
**Academic Stress**	0.68(0.60 to 0.76)	16.467	< 0.001
**Coronaphobia**	0.51(0.43 to 0.60)	12.119	< 0.001
**Knowledge**	-0.02(-0.18 to 0.13)	-0.301	0.764
**Precautionary measures**	-0.34(-0.55 to -0.12)	-3.051	0.002

**Note.** This table showed the output from a multivariable regression analysis in which generalized anxiety mean score differences across the study was the dependent variable. Overall fit of the model was *R*^2^ = 0.500, *P* < 0.001. Enter regression method was used. Gender (0 = men, 1 = women), Grade (0 = Grade 5–6, 1 = Grade 7–8), Shantou/Hezhou (0 = Nanchong, 1 = Shantou/Hezhou), Father’s education level/Mother’s education level (1 = Under junior high school, 2 = Senior school, 3 = Undergraduate, 4 = Master degree or above), Father’s occupation/Mother’s occupation (0 = non-Health worker, 1 = Health worker), Whether lives with parents (0 = Neither, 1 = lives with father/mother, 2 = lives with father and mother), Low-income family (0 = no, 1 = yes). Variate that was filled by respondent eliminated who chose “Unknown” was marked with a pound sign (^#^) after the name

### Linear regression analysis by city

Table 3 presented standardized coefficients estimates for influencing factors by anxiety and by city. Generalized anxiety was positively related to academic stress and coronaphobia by city. Associations of anxiety with academic stress was stronger than those between anxiety and coronaphobia in each rural area. A negative association between generalized anxiety and precautionary measures was observed in Hezhou and Nanchong, with the exception of Shantou.

**Table 3:** Adjusted Standardized Coefficients Estimates of the Influencing Factors for Generalized Anxiety by City

Variates	Generalized anxiety		
	Shantou, Guangdong Beta	Hezhou, Guangxi Beta	Nanchong, Sichuan Beta
**Academic Stress**	0.506**	0.464**	0.493**
**Coronaphobia**	0.321**	0.429**	0.314**
**Precautionary measures**	-0.039	-0.122**	-0.083*

**Note.** This table showed the output of multivariable regression analyses among cities, in which generalized anxiety average score differences were the dependent variables. Fit of the adjusted models were *R*^2^ = 0.501, *P* < 0.001 (Shantou), *R*^2^ = 0.540, *P* < 0.001 (Hezhou) and *R*^2^ = 0.436, *P* < 0.001 (Nanchong). “Beta” is the standardized regression coefficient. Enter regression method was used. Statistical significances by city were represented in table by asterisks as follows: * < 0.05; ** < 0.01

## Discussion

As the pandemic continues, mental health has become another important issue besides physical health. People continuously exposed to the adverse outbreak-related information might suffer from depression, anxiety, and stress disorders [26]. The present multicenter study revealed that the prevalence of anxiety among rural primary and middle school students in southern China during the outbreak of COVID-19 period was 33.8% (Shantou, Guangdong: 30.0%, Hezhou, Guangxi: 37.9%, Nanchong, Sichuan: 34.3%). The prevalence of anxiety in adolescents was higher than that (19.0%) [27] in initial outbreak of the epidemics. Moreover, the prevalence of anxiety in adolescents was reported to be higher than the global prevalence reported for adolescents (20.5%) [28]. The possible reasons for this phenomenon were as follows: First, prevalence rates may vary from study to study-conducting period. The afore-mentioned studies were performed mainly in the initial phase of the outbreak, whereas our study was conducted during the ongoing COVID-19 period. During the ongoing outbreak, people might be more concern about when it could be terminated. Meanwhile, our study was conducted when students resumed school. The discomfort of students learning online before returning to school led to the academic stress of returning to school, which might have contributed to this. Second, compared with urban students, rural students might be more sensitive and vulnerable to the mental effects of the outbreak due to the lack of psychological guidance. This might be why they were more prone to anxiety during this period. Thus, more targeted measures should be implemented for rural students.

In addition, inequality is a growing problem in rural China, where the economy, social structure, and consumption pattern have changed dramatically [29]. Province was a dominant contributor to anxiety inequality for rural students, suggesting that the relevant effects (including environmental effects) vary considerably across provinces. Results of linear regression analysis by rural area revealed that, during part of the COVID-19 epidemic, anxiety was strongly associated with academic stress and coronaphobia. Furthermore, precautionary measure was a risk factor for anxiety among rural students in Hezhou and Nanchong.

Regarding academic stress, students who were worried about academics reported higher risks of anxiety than students with less pressure of study. The outbreak of COVID-19 quickly spread to various places in China [30], coinciding with the Chinese Lunar New Year. In both urban and rural areas, the local government acted quickly and formulated some effective measures, such as encouraging people to stay at home and delaying school return [31]. Teachers in rural areas have also taught online through the Internet. Although nationwide efforts have been made to mitigate disruptions of learning, the quarantine and online learning might negatively impact rural students’ educational outcomes. Firstly, online learning was not feasible in many rural households due to the lack of appropriate electronic devices. To be able to study online, a large proportion of students had to use mobile phones, and very few use computers [32]. Secondly, local teachers teaching online were unable to see students in their classes, which seemed to be a critical lack of student-teacher interaction [32]. Even some students turned to asynchronous learning by watching educational television broadcasts, for which they were instructed to tune into a specific channel to watch videos of pre-recorded lectures. Online classes would decrease the effectiveness of student-teacher interactions, which have been shown to be detrimental to students’ learning 33. Finally, the children’s education might be attributed in part to family environment. The guardians of most rural students, mainly grandparents and even some rural parents, had limited education and were not familiar enough with online learning technology, which drastically restricted their ability to help children with online assignments [34]. These problems can easily lead to academic stress for rural students, which leads to their anxiety.

Our study also demonstrated that students with coronaphobia suffered from general anxiety and that having coronaphobia posed a major risk factor for this form of psychopathology. For example, with coronaphobia, people appeared to be afraid that he acquired the disease so they suffered from psychological problems [22, 35] and even commit suicide [36]. These findings also highlighted the need for rural teachers to concern the psychological status of students, giving them mental persuasion and support, as it not only was linked to mental distress, but it may be the underlying cause of other physical and mental complaints during this epidemic [37].

For Hezhou and Nanchong, the better the comprehensive preventive measures were, the less likely people were to be anxious. According to the COVID-19 prevention guidebook by WHO [25], people should wear masks, wash hands frequently and do more exercise as our preventive measures, which have protective effects on psychological status. Mask-wearing, for instance, is widely believed to prevent patients from spreading viruses while also protecting the wearer from infection [38], as well as relieving anxiety and depression [26]. Moreover, regular physical activities regarding intensities and forms could be associated with lower levels of depression and anxiety [39, 40].

As the COVID-19 outbreak is no longer confined to large cities, issues related to school closures and home isolation are also becoming important in rural areas. In addition, rural children have few voices to advocate for their needs. Ensuring that the physical and psychological impact of the COVID-19 outbreak on children and adolescents is minimized is the responsibility and vital interest of all stakeholders, from the government to parents. Immediate action must be taken. Sustainable programs must involve local professionals, culturally adapt interventions to administrative systems and regional and community settings, and contextually relevant materials must be developed for children and adolescents.

Our findings would inform policy decisions to monitor and support the mental health of rural students. Data from multicenter sources (12 schools in Shantou, Hezhou, and Nanchong) and large sample size (1179 respondents) improved the generalizability of rural students in southern China. In addition, we were concerned that a few rural students did not have their own Internet devices or that they might not understand parts of the questionnaire. Therefore, unlike most online surveys done during the epidemic, our survey was conducted by local trained investigators who presented the relevant content of this study and distributed and received paper questionnaires on the spot, which may have reduced selection bias and increased the reliability of the answers.

Nevertheless, this study also has some limitations that are important to note. First, the cross-sectional design of the study mitigates our ability to infer causal relationships. Second, because of the sudden outbreak, baseline data on the mental health status of the target population during normal conditions without an epidemic were not available so that excess morbidity of anxiety due to the epidemic could not be correctly determined. Thus, coronaphobia, COVID-19 knowledge and precautionary measures in this study were analyzed the relationship with general anxiety to study impact of COVID-19 on anxiety, as we discussed above. Third, the rural areas of Guangdong, Guangxi and Sichuan were chosen as the three main southern rural areas to represent Chinese other rural areas, excluding urban areas. This might produce a biased sample that cannot adequately represent the entire Chinese adolescent population.

## Conclusion

In conclusion, the COVID-19 outbreak has had a significant psychosocial impact on rural children and adolescents. The adolescents with academic stress and coronaphobia, the greater the risk that adolescents will suffer from anxiety, suggesting mental health counseling and professional family support are needed.

## Data Availability

All of the data generated or analysed during this study are included in this published article.

## References

[1] Bao Y, Sun Y, Meng S, Shi J, Lu L (2020). 2019-nCoV epidemic: address mental health care to empower society. Lancet Lond Engl.

[2] Xu XW, Wu XX, Jiang XG, Xu KJ, Ying LJ, Ma CL et al. Clinical findings in a group of patients infected with the 2019 novel coronavirus (SARS-Cov-2) outside of Wuhan, China: retrospective case series.BMJ. 2020;368:m606. 10.1136/bmj.m606 PMC7224340 32075786

[3] WHO. WHO Coronavirus (COVID-19) Dashboard. 2020. https://covid19.who.int. Accessed 21 Nov 2021.

[4] Heymann DL, Shindo N (2020). COVID-19: what is next for public health?. The Lancet.

[5] Lancet T (2020). COVID-19 and China: lessons and the way forward. The Lancet.

[6] Brooks SK, Webster RK, Smith LE, Woodland L, Wessely S, Greenberg N (2020). The psychological impact of quarantine and how to reduce it: rapid review of the evidence. The Lancet.

[7] CCTV. National Health Commission of the People’s Republic of China: All provinces in China lowered the public health emergency response to Level 2 or lower. 2020. http://news.cctv.com/2020/05/02/ARTIJLXYsSmI40i4URDzFtXi200502.shtml. Accessed 21 Nov 2021.

[8] Huang Y, Wang Y, Wang H, Liu Z, Yu X, Yan J (2019). Prevalence of mental disorders in China: a cross-sectional epidemiological study. Lancet Psychiatry.

[9] Zhang L, Xu Y, Nie H, Zhang Y, Wu Y (2012). The prevalence of depressive symptoms among the older in China: a meta-analysis: the prevalence of depressive symptoms. Int J Geriatr Psychiatry.

[10] Guo J, Fu M, Liu D, Zhang B, Wang X, van IJzendoorn MH (2020). Is the psychological impact of exposure to COVID-19 stronger in adolescents with pre-pandemic maltreatment experiences? A survey of rural chinese adolescents. Child Abuse Negl.

[11] Larsen B, Luna B (2018). Adolescence as a neurobiological critical period for the development of higher-order cognition. Neurosci Biobehav Rev.

[12] Blakemore S-J (2019). Adolescence and mental health. The Lancet.

[13] de Figueiredo CS, Sandre PC, Portugal LCL, Mázala-de-Oliveira T, da Silva Chagas L, Raony Í (2021). COVID-19 pandemic impact on children and adolescents’ mental health: Biological, environmental, and social factors. Prog Neuropsychopharmacol Biol Psychiatry.

[14] Johnson SU, Ulvenes PG, Øktedalen T, Hoffart A (2019). Psychometric Properties of the General anxiety disorder 7-Item (GAD-7) scale in a Heterogeneous Psychiatric Sample. Front Psychol.

[15] Spitzer RL, Kroenke K, Williams JBW, Löwe B (2006). A brief measure for assessing generalized anxiety disorder: the GAD-7. Arch Intern Med.

[16] He X, Li C, Qian J, Cui H, Wu W (2010). Reliability and validity of a generalized anxiety disorder scale in general hospital outpatient. Shanghai Arch Psychiatry Shanghai Arch Psychiatry.

[17] Engel ML, Shanley R, Scal PB, Kunin-Batson A (2021). Anxiety and depressive symptoms in adolescents and young adults with epilepsy: the role of illness beliefs and social factors. Epilepsy Behav.

[18] Mossman SA, Luft MJ, Schroeder HK, Varney ST, Fleck DE, Barzman DH (2017). The generalized anxiety disorder 7-item (GAD-7) scale in adolescents with generalized anxiety disorder: signal detection and validation. Ann Clin Psychiatry Off J Am Acad Clin Psychiatr.

[19] Wang X, Hegde S, Son C, Keller B, Smith A, Sasangohar F (2020). Investigating Mental Health of US College Students during the COVID-19 Pandemic: cross-sectional survey study. J Med Internet Res.

[20] Yan Y. A case study of social work intervention in college students’ learning anxiety. Beijing University Of Technology; 2019.

[21] Asmundson GJG, Taylor S (2020). Coronaphobia: fear and the 2019-nCoV outbreak. J Anxiety Disord.

[22] Lee SA (2020). Coronavirus anxiety scale: a brief mental health screener for COVID-19 related anxiety. Death Stud.

[23] Lee SA, Jobe MC, Mathis AA, Gibbons JA (2020). Incremental validity of coronaphobia: coronavirus anxiety explains depression, generalized anxiety, and death anxiety. J Anxiety Disord.

[24] Chinese Center for Disease Control and Prevention. Circular of the General Office of the National Health Commission on the issuance of COVID-19 Prevention and Control Plan (fifth edition). 2020. http://www.nhc.gov.cn/jkj/s3577/202002/a5d6f7b8c48c451c87dba14889b30147.shtml. Accessed 21 Nov 2021.

[25] World Health Organization. Advice for the public. 2020. https://www.who.int/emergencies/diseases/novel-coronavirus-2019/advice-for-public. Accessed 21 Nov 2021.

[26] Wang C, Pan R, Wan X, Tan Y, Xu L, McIntyre RS (2020). A longitudinal study on the mental health of general population during the COVID-19 epidemic in China. Brain Behav Immun.

[27] Qi H, Liu R, Chen X, Yuan X, Li Y, Huang H (2020). Prevalence of anxiety and associated factors for chinese adolescents during the COVID -19 outbreak. Psychiatry Clin Neurosci.

[28] Racine N, McArthur BA, Cooke JE, Eirich R, Zhu J, Madigan S (2021). Global prevalence of depressive and anxiety symptoms in children and adolescents during COVID-19: a Meta-analysis. JAMA Pediatr.

[29] Wiedenhofer D, Guan D, Liu Z, Meng J, Zhang N, Wei Y-M (2017). Unequal household carbon footprints in China. Nat Clim Change.

[30] National Health Commission of the People’s Republic of China. Updates on the epidemic. 2020. http://www.nhc.gov.cn/xcs/yqtb/list_gzbd.shtml. Accessed 21 Nov 2021.

[31] Fu B, Fu X (2020). The model of epidemic (COVID-19) prevention and control in rural of China. Crit Care.

[32] Wang H, Zhang M, Li R, Zhong O, Johnstone H, Zhou H (2021). Tracking the effects of COVID-19 in rural China over time. Int J Equity Health.

[33] Bettinger E, Loeb S. Promises and pitfalls of online education. 2017;2:1-4.

[34] Zeng Z, Xie Y (2014). The Effects of grandparents on children’s schooling: evidence from Rural China. Demography.

[35] Lai J, Ma S, Wang Y, Cai Z, Hu J, Wei N (2020). Factors Associated with Mental Health Outcomes among Health Care Workers exposed to Coronavirus Disease 2019. JAMA Netw Open.

[36] Goyal K, Chauhan P, Chhikara K, Gupta P, Singh MP (2020). Fear of COVID 2019: first suicidal case in India !. Asian J Psychiatry.

[37] Taylor S. The psychology of pandemics: preparing for the Next Global Outbreak of Infectious Disease. Cambridge Scholars Publishing; 2019.

[38] Kim S-W, Su K-P (2020). Using psychoneuroimmunity against COVID-19. Brain Behav Immun.

[39] Hamer M, Stamatakis E, Steptoe A (2009). Dose-response relationship between physical activity and mental health: the Scottish Health Survey. Br J Sports Med.

[40] Peçanha T, Goessler KF, Roschel H, Gualano B (2020). Social isolation during the COVID-19 pandemic can increase physical inactivity and the global burden of cardiovascular disease. Am J Physiol-Heart Circ Physiol.

